# Improving the Precision of Etiological Diagnosis in Bacterial Infections Using Molecular Technologies: A Comparative Analysis of Platforms, AI Integration, and Point-of-Care Deployment

**DOI:** 10.3390/ijms27094148

**Published:** 2026-05-06

**Authors:** Alina-Marinela Cotelici, Andrei Theodor Bălășoiu, Mihail Virgil Boldeanu, Mohamed-Zakaria Assani, Marius Bogdan Novac, Lidia Boldeanu, Alice Elena Ghenea

**Affiliations:** 1 Doctoral School, University of Medicine and Pharmacy of Craiova, 200349 Craiova, Romania; alina.cotelici@yahoo.com (A.-M.C.); mohamed.assani@umfcv.ro (M.-Z.A.); 2 Department of Otorhinolaryngology, University of Medicine and Pharmacy of Craiova, 200349 Craiova, Romania; andrei_theo@yahoo.com; 3 Department of Immunology, Faculty of Medicine, University of Medicine and Pharmacy of Craiova, 200349 Craiova, Romania; mihail.boldeanu@umfcv.ro; 4 Department of Anesthesiology and Intensive Care, University of Medicine and Pharmacy of Craiova, 200349 Craiova, Romania; 5 Department of Microbiology, Faculty of Medicine, University of Medicine and Pharmacy of Craiova, 200349 Craiova, Romania; gaman_alice@yahoo.com

**Keywords:** molecular diagnostics, bacterial infections, PCR, next-generation sequencing, CRISPR diagnostics, antimicrobial resistance, point-of-care, machine learning, comparative performance, precision microbiology

## Abstract

Bacterial infections remain a major global health burden, further exacerbated by the rapid emergence of antimicrobial resistance (AMR), which increases the need for accurate and timely etiological diagnosis. Conventional culture-based methods are limited by prolonged turnaround times, reduced sensitivity in patients receiving prior antimicrobial therapy, and restricted ability to characterize resistance mechanisms at the molecular level. Molecular diagnostic technologies have significantly transformed bacteriological diagnostics by enabling rapid, sensitive, and specific pathogen detection directly from clinical specimens. This review provides a structured comparative analysis of major molecular platforms, including polymerase chain reaction (PCR) and its variants, isothermal amplification technologies, next-generation sequencing (NGS), clustered regularly interspaced short palindromic repeats (CRISPR) based diagnostics, and digital PCR (dPCR). Key analytical parameters such as sensitivity, specificity, limit of detection (LOD), time to result, and multiplexing capacity are evaluated to highlight platform-specific strengths and limitations. In addition, the integration of artificial intelligence and machine learning (AI/ML) into molecular diagnostic workflows for AMR prediction and clinical decision support is critically examined. The translational potential of these technologies toward point-of-care (POC) implementation is also discussed, with consideration of clinical validation, operational constraints, and real-world applicability. Overall, this review provides an integrated perspective on current molecular diagnostic strategies, emphasizing the balance between analytical performance and clinical interpretability, and outlines key challenges and future directions for advancing culture-independent bacteriological diagnostics.

## 1. Introduction

Bacterial infections represent a leading cause of morbidity and mortality worldwide. A 2022 landmark analysis estimated that 33 bacterial pathogens were directly responsible for 7.7 million deaths in 2019, positioning bacterial infections as the second leading cause of global mortality [1]. The progressive dissemination of multidrug-resistant (MDR) and extensively drug-resistant (XDR) strains has further amplified the clinical imperative for diagnostic strategies. This trend is driven by multiple factors, including inappropriate antibiotic prescribing, inadequate infection control, and increased global travel [2].

Accurate and timely etiological diagnosis is a foundational determinant of clinical outcome. The diagnostic gap, the interval between clinical suspicion of infection and receipt of actionable microbiological results, is estimated at 48–96 h under conventional culture-based workflows. During this period, empirical broad-spectrum antimicrobial therapy is initiated in the absence of microbiological data, contributing to inappropriate prescribing in 20–40% of cases, accelerated emergence of resistance, and preventable adverse outcomes [3]. In sepsis, where each hour of untargeted therapy is independently associated with increased mortality, such delays are clinically untenable [4].

Molecular diagnostic technologies, encompassing nucleic acid amplification tests (NAATs), next-generation sequencing (NGS), and emerging CRISPR-based and digital platforms, have substantially narrowed this diagnostic gap. Nevertheless, the clinical translation of these technologies into routine practice remains heterogeneous, constrained by analytical complexity, interpretive challenges, regulatory barriers, and cost–benefit considerations [5].

While previous reviews have addressed individual molecular platforms or specific infection syndromes, no existing publication provides a unified comparative framework that simultaneously benchmarks platform performance quantitatively, evaluates artificial intelligence and machine learning (AI/ML) integration as an analytical enabler, and assesses the translational readiness of each technology for point-of-care (POC) deployment. 

This review provides a structured analysis of current molecular diagnostic strategies for bacterial pathogens, focusing on analytical performance, detection of antimicrobial resistance (AMR), and clinical applicability. First, we present a comparative evaluation of established and emerging molecular platforms, including polymerase chain reaction (PCR) based methods, sequencing technologies, and clustered regularly interspaced short palindromic repeats (CRISPR) based diagnostics. Second, we examine the integration of AI in molecular diagnostics, with emphasis on AMR prediction and data interpretation. Third, we assess the translation of these technologies into POC settings, highlighting implementation challenges and opportunities.

This review is based on a narrative synthesis of the literature. Relevant studies were identified through searches of major scientific databases, including PubMed, Scopus, and Web of Science, covering publications up to early 2026. Search strategies combined keywords related to molecular diagnostics, AMR, AI, CRISPR-based detection, and nanopore sequencing. Particular emphasis was placed on recent studies addressing rapidly evolving technologies, such as AI/ML and CRISPR-based diagnostics.

Both primary research articles and high-quality review papers were considered, with preference given to studies reporting analytical performance, clinical validation, or real-world implementation data. When multiple studies were available, priority was given to systematic reviews, meta-analyses, and multicenter evaluations to ensure robustness of the comparative framework. Earlier references were included where they represent foundational contributions or widely cited validation studies that remain relevant for contextualizing current platform capabilities.

## 2. Conventional Microbiological Diagnostics: The Diagnostic Gap

The conventional diagnostic algorithm for bacterial infections, encompassing specimen collection, direct microscopy, culture-based pathogen recovery, MALDI-TOF MS identification, and phenotypic antimicrobial susceptibility testing (AST), has defined the microbiological standard of care for over a century. MALDI-TOF MS has substantially accelerated organism identification from isolated colonies, reducing identification time to minutes with species-level accuracy exceeding 95% for most clinically relevant bacteria [6]. Nevertheless, MALDI-TOF MS is contingent upon successful culture, inheriting the temporal and sensitivity limitations of the culture step.

Phenotypic AST on automated platforms (VITEK 2, MicroScan, Phoenix) requires pure culture and provides categorical susceptibility results in 8–16 h under optimal conditions. However, these platforms are susceptible to categorical error for organisms exhibiting heteroresistance or complex resistance mechanisms, notably carbapenem-resistant Enterobacterales (CRE), where phenotypic interpretive categories may fail to reflect the clinical significance of underlying resistance genes [7].

The cumulative diagnostic gap under conventional workflows is 48–96 h for blood culture-based bacteremia workups and may exceed five days for fastidious organisms or specimens from patients with prior antibiotic exposure. Epidemiological studies indicate that this gap translates directly into excess mortality, longer hospitalizations, and broader antimicrobial selection pressure, forming the primary clinical justification for the adoption of molecular diagnostics [3,4].

## 3. Molecular Diagnostic Platforms: Overview and Analytical Performance

### 3.1. PCR-Based Technologies

#### 3.1.1. Real-Time Quantitative PCR (qPCR)

Quantitative PCR (qPCR), incorporating TaqMan probe hydrolysis or SYBR Green intercalation chemistry, enables simultaneous amplification and quantification of pathogen-specific nucleic acid targets with clinical sensitivity and specificity frequently exceeding 95% and 98%, respectively, for well-validated bacterial targets [8]. For MRSA detection from clinical specimens, qPCR-based assays demonstrate pooled sensitivity of 93.5% and specificity of 97.8% across multiple meta-analytic datasets, with a time to result of 1–3 h—a performance profile fundamentally superior to culture-based MRSA screening [9]. However, an important limitation of qPCR-based detection is that nucleic acid amplification does not discriminate between viable and nonviable organisms. In clinical contexts such as sepsis or in patients receiving prior antimicrobial therapy, pathogen-derived cell-free DNA may persist in circulation for several days despite microbiological clearance. This may lead to positive qPCR results in the absence of active infection, thereby reducing clinical specificity and complicating interpretation. In this regard, RNA-targeting approaches such as transcription-mediated amplification (TMA) may provide improved correlation with microbial viability, as further discussed in Section 3.2.

For resistance gene detection, qPCR targeting mecA/mecC, vanA/B, blaKPC, blaNDM, blaOXA-48, and blaVIM achieves analytical sensitivity in the range of 1–100 genome equivalents per reaction, enabling detection in specimens with low organism loads [10].

#### 3.1.2. Multiplex PCR Syndromic Panels

Multiplex PCR platforms—including BioFire FilmArray (bioMérieux), Seegene Allplex, and Hologic Panther Fusion—simultaneously interrogate panels of 10 to more than 40 bacterial targets, along with resistance markers, in a single reaction. The FilmArray Blood Culture Identification Panel 2 (BCID2), encompassing 43 pathogen and resistance targets including mecA, vanA/B, blaKPC, blaNDM, and blaOXA-48-like, demonstrated 96.5% overall concordance with conventional culture-based identification in a multicenter evaluation, with a median time to result of 73 min from positive blood culture flag [11]. Prospective intervention studies demonstrated that, when panel results were coupled with structured antimicrobial stewardship programs, appropriate antimicrobial therapy was initiated 25 h earlier, with associated reductions in 30-day mortality among bacteremic patients [12].

#### 3.1.3. Digital PCR (dPCR)

Digital PCR partitions nucleic acid samples into thousands of individual reaction chambers, enabling absolute quantification through Poisson statistical analysis of binary readouts, with a limit of detection one to two orders of magnitude lower than conventional qPCR. Droplet digital PCR (ddPCR) has demonstrated utility in detecting low-level carbapenemase gene copies below the qPCR threshold in surveillance specimens, with direct implications for outbreak prevention [13]. Clinical applications include precise quantification of pathogen burden for therapeutic monitoring and detection of heteroresistance in specimens from patients with prior antibiotic exposure.

### 3.2. Isothermal Nucleic Acid Amplification

Loop-Mediated Isothermal Amplification (LAMP) employs a strand-displacing polymerase at a constant temperature (60–65 °C), yielding results in 15–60 min without thermocycling equipment. A systematic review and meta-analysis of LAMP for M. tuberculosis detection demonstrated a pooled sensitivity of 97.7% and a specificity of 99.1% in smear-positive specimens [14]. 

Colorimetric LAMP for *C. difficile* toxin gene detection achieved a sensitivity of 94.3% and a specificity of 97.8% compared with PCR reference in stool specimens, with a cost per test substantially below that of syndromic panel platforms [15]. 

Recombinase Polymerase Amplification (RPA) amplifies at near-physiological temperature (37–42 °C), while TMA, selective for RNA targets, including ribosomal RNA, enables detection of viable organisms and discrimination from dead bacteria, a critical feature for therapeutic monitoring [16]. This distinction is particularly relevant in the context of DNA-based amplification methods such as qPCR, where detection of nucleic acids does not necessarily indicate the presence of viable organisms, potentially affecting clinical interpretability.

### 3.3. Next-Generation Sequencing (NGS)

#### 3.3.1. Whole-Genome Sequencing (WGS)

WGS using Illumina short-read or Oxford Nanopore long-read platforms provides comprehensive genomic characterization of bacterial isolates—encompassing species identification, multi-locus sequence typing (MLST), whole-genome phylogenetics, complete characterization of the resistome and virulome, and determination of plasmid architecture [17]. Meta-analytic data demonstrate WGS resistome–phenotype concordance exceeding 95% for major antibiotic classes in *Staphylococcus aureus*, *Enterococcus* spp., and *Enterobacterales* [18]. WGS has been established as the cornerstone of national bacterial surveillance programs in multiple European countries and the United States, providing near-real-time phylogeographic tracking of priority pathogens [19].

Recent genomic surveillance frameworks further reinforce the central role of WGS in real-time pathogen characterization and AMR tracking across clinical, environmental, and one health settings. In particular, large-scale integrative approaches highlight WGS’s capacity to support high-resolution phylogenetic analysis, outbreak detection, and cross-sectoral transmission mapping, thereby enabling near-real-time epidemiological insights at local, national, and global scales. These frameworks emphasize not only the analytical power of genomic data but also the importance of standardized bioinformatic pipelines, interoperable databases, and coordinated data-sharing infrastructures. Furthermore, the integration of genomic surveillance into routine public health systems is increasingly recognized as a cornerstone of effective AMR monitoring and response strategies, particularly for emerging multidrug-resistant pathogens. Together, these developments position WGS as a foundational technology bridging clinical microbiology and public health surveillance in the era of precision infectious disease management [20].

#### 3.3.2. Metagenomic NGS (mNGS)

Metagenomic NGS enables untargeted, culture-independent pathogen identification from clinical specimens by aligning all sequenced nucleic acids to comprehensive microbial reference databases. A prospective multicenter study of patients with unexplained sepsis demonstrated that mNGS pathogen identification was achieved in 52.9% of culture-negative cases, with a direct impact on clinical management in 29.4% [21]. For central nervous system infections—the application where mNGS evidence is most robust—Wilson et al. demonstrated CSF mNGS identified the causative agent in 13% of cases with negative conventional diagnostics, including rare and unexpected pathogens [22]. However, mNGS faces implementation barriers: the low fraction of microbial reads in blood (<0.01%), background contamination, complex interpretive requirements, and current per-test costs of USD 250–2000 limit routine deployment to specialized settings [23].

#### 3.3.3. Nanopore-Based Rapid Sequencing

Oxford Nanopore Technologies (ONT) MinION enables real-time, long-read sequencing with clinical bacteremia turnaround—from positive blood culture to comprehensive genomic report—of as little as 6 h in optimized workflows. A prospective nanopore-targeted sequencing (NTS) study analyzing 387 blood samples demonstrated high concordance with mNGS and an 80.6% genotype–phenotype correlation for resistance genes in bloodstream infections, with species identification achievable within 30 min of sequencing initiation [24]. 

Recent clinical studies further support the utility of nanopore sequencing for rapid pathogen identification in sterile body fluids and central nervous system infections [25,26].

### 3.4. CRISPR-Based Diagnostic Platforms

The CRISPR-based diagnostics exploit the programmable target recognition of guide RNAs, combined with the trans-cleavage (collateral nuclease) activity of Cas12 and Cas13 effectors, to generate sequence-specific signals. The SHERLOCK platform (Cas13a-mediated RNA detection with lateral flow readout) achieves attomolar sensitivity and enables single-nucleotide variant discrimination without high-resolution melting curve analysis, enabling differentiation of resistance alleles [27]. The DETECTR system (Cas12a with lateral flow) achieved 100% concordance with PCR for the detection of the C. difficile toxin gene in a prospective clinical evaluation [28]. Recent advances include CRISPR-Cas12a microfluidic chip systems that simultaneously identify eight pathogens in 45 min, with 99.2% concordance with qPCR, and are currently undergoing CE marking validation [29].

For AMR detection specifically, CRISPR-based assays targeting blaKPC, blaNDM, and mecA variants have demonstrated single-nucleotide discriminatory capacity that PCR-based platforms cannot achieve without supplementary high-resolution melt analysis [30]. A CRISPR/Cas13a-based simultaneous amplification and testing platform (SATCAS), integrating one-pot RNA detection and SNP discrimination, was clinically validated in 2024 [31].

Despite their remarkable analytical performance, the clinical translation of CRISPR-based diagnostics remains an evolving process. Recent advances have demonstrated that CRISPR systems, particularly Cas12 and Cas13, enable highly sensitive and specific nucleic acid detection through programmable target recognition coupled with collateral cleavage-based signal amplification, including the ability to discriminate single-nucleotide variants relevant to antimicrobial resistance profiling. However, beyond analytical performance, emerging evidence highlights several translational challenges that currently limit widespread clinical adoption, including variability in assay standardization, constraints in large-scale manufacturing, and the need for robust integration into routine diagnostic workflows and regulatory frameworks [32]. Furthermore, issues related to reproducibility across platforms and the requirement for clinical validation in diverse real-world settings remain critical barriers. Collectively, while CRISPR-based diagnostics represent one of the most promising next-generation molecular platforms, their successful implementation will depend on addressing these translational and operational challenges alongside continued technological refinement.

Recent developments, including droplet digital CRISPR and electrochemiluminescence-based platforms, have further expanded the sensitivity and translational potential of CRISPR diagnostics [33,34].

## 4. Comparative Performance Analysis of Molecular Platforms

Table 1 provides a structured side-by-side comparison of major molecular diagnostic platforms for etiological diagnosis of bacterial infections across seven standardized analytical parameters. This comparative framework, absent from existing reviews, enables direct clinical and laboratory decision-making regarding platform selection based on context-specific performance requirements.

The performance metrics summarized in Table 1 were derived from a narrative synthesis of the literature, prioritizing systematic reviews and meta-analyses for aggregated performance estimates, complemented by representative primary studies and, where available, representative multicenter validation studies. Where possible, more recent studies were preferentially considered to reflect current platform capabilities, while earlier references were retained when representing foundational or widely cited validation data. Reported values for sensitivity, specificity, and limit of detection (LOD) reflect ranges observed across multiple studies rather than single-study estimates and were harmonized by grouping comparable analytical conditions across platforms.

Given the heterogeneity of study designs, specimen types, and analytical methodologies, direct comparison of certain parameters—particularly LOD values—should be interpreted with caution. Differences in reporting units (e.g., copies per reaction, copies per µL, or molar concentration) and experimental conditions may affect comparability across technologies.

Furthermore, the evidence base spans multiple technological generations; therefore, reported performance ranges reflect aggregated values rather than the capabilities of the most recent platform iterations. This is particularly relevant for rapidly evolving technologies such as CRISPR-based diagnostics, nanopore sequencing, and AI-assisted molecular workflows, where ongoing advancements may exceed the ranges summarized here.

Several key observations emerge from this comparative analysis. First, there is an inherent performance trade-off between analytical comprehensiveness and time to result: WGS and mNGS provide the most complete pathogen characterization but require 12–72 h and are currently confined to centralized laboratories. LAMP, RPA, and CRISPR-based platforms offer the shortest turnaround times (15–60 min) and highest POC feasibility, but at the cost of reduced multiplexing breadth. Second, digital PCR occupies a distinct niche defined by unmatched quantitative precision and sensitivity but is not suited to broad syndromic diagnostics or POC contexts. Third, CRISPR-based platforms are unique in combining attomolar sensitivity with single-nucleotide discrimination and POC-compatible lateral-flow readout, positioning them as the most likely candidates for next-generation decentralized precision AMR diagnostics.

The selection of the optimal platform in clinical practice is therefore context-dependent, and a tiered diagnostic algorithm—deploying rapid POC platforms for initial triage and reserving NGS-based approaches for complex, culture-negative, or epidemiologically significant cases—represents the most clinically and economically rational strategy [5,35].

## 5. Molecular AMR Genotyping: Current Capabilities and Limitations

Molecular AMR genotyping—the detection of resistance determinants at the nucleic acid level—provides actionable resistance information within timeframes compatible with clinical decision-making, independent of the culture-based AST workflows that currently impose the critical diagnostic delay in bacteremia management. The principal platforms for AMR genotyping span a spectrum from targeted single-gene assays to comprehensive whole-genome resistome profiling.

Targeted PCR-based AMR detection panels—encompassing mecA/mecC for MRSA, vanA/B for VRE, blaKPC, blaNDM, blaOXA-48-like, blaVIM, and blaIMP for carbapenemase-producing organisms (CPOs)—represent the most clinically mature and widely deployed molecular AMR tools. A prospective multicenter evaluation of Xpert Carba-R for detection of carbapenemase genes in rectal surveillance swabs demonstrated a sensitivity of 98.1% and specificity of 99.5% compared with culture and confirmatory PCR, with a time to result of 75 min [36]. These performance parameters are clinically sufficient for infection control decision-making and have been incorporated into national CPO surveillance guidelines in multiple European countries.

WGS-based comprehensive resistome profiling, interrogating curated databases including Comprehensive Antibiotic Resistance Database (CARD), ResFinder, ARG-ANNOT, and NCBI AMRFinder Plus, provides genotype–phenotype concordance exceeding 95% for major antibiotic classes in Enterobacterales and *S. aureus* [18]. However, significant concordance gaps persist for organisms with novel or chromosomally mediated resistance mechanisms, for which phenotypic reference databases may lag behind the emergence of clinical resistance. The 2021 WHO catalog of M. tuberculosis mutations associated with drug resistance, integrating data from 38,215 isolates, established the most comprehensive genotype–phenotype framework to date for a single pathogen, achieving sensitivity of 94.3% and specificity of 99.4% for rifampicin resistance prediction [37].

A critical limitation of genotypic AMR prediction is the inherent database dependency: resistance genes or mutations not represented in the reference database will not be detected, creating a systematic blind spot for novel mechanisms. Furthermore, the detection of a resistance gene does not invariably predict phenotypic resistance, as expression-level regulation, gene copy number variation, and epistatic interactions between resistance determinants may attenuate or enhance phenotypic expression beyond what genotype alone can predict [38].

Clinically relevant genotype–phenotype discordance remains a critical limitation of molecular AMR prediction. In several well-documented scenarios, the presence of a resistance gene does not translate into phenotypic resistance. For example, mecA-positive Staphylococcus aureus isolates may remain phenotypically susceptible due to low or heterogeneous gene expression, whereas vanA-positive Enterococcus strains may exhibit variable resistance depending on activation of regulatory genes. Conversely, phenotypic resistance may occur in the absence of canonical resistance genes, particularly in Gram-negative pathogens such as Pseudomonas aeruginosa or Acinetobacter baumannii, where resistance is frequently mediated by chromosomal mutations affecting porin expression, efflux pump upregulation, or target site modification, mechanisms that are not consistently captured by targeted molecular assays [18,38]. Furthermore, emerging or rare resistance determinants not yet included in reference databases represent an additional source of discordance in sequencing-based approaches [38,39]. From a clinical perspective, such discrepancies may lead to inappropriate antimicrobial selection if genotypic data are interpreted in isolation. Consequently, while molecular AMR detection provides rapid and actionable insights, it cannot fully replace phenotypic susceptibility testing, which remains essential for capturing the functional expression of resistance mechanisms in routine clinical practice [18,40].

These limitations are also highly relevant to AI-based AMR prediction models, which rely on genomic features and may therefore inherit the same genotype–phenotype discrepancies discussed above (see Section 6).

## 6. Artificial Intelligence and Machine Learning in Molecular Bacteriological Diagnostics

The integration of artificial intelligence (AI) and machine learning into molecular diagnostic workflows represents one of the most consequential developments in contemporary clinical microbiology. AI/ML applications address critical bottlenecks in interpreting data-rich molecular platforms, particularly WGS, mNGS, and multiplex panels, where the volume and complexity of generated data preclude efficient manual analysis at clinical-throughput scales [41].

AI is increasingly integrated into clinical microbiology laboratories, contributing to improved diagnostic accuracy, automated data interpretation, and enhanced workflow efficiency. Beyond analytical performance, AI applications are expanding into broader clinical and operational domains, including decision support, epidemiological analysis, and mitigating workforce limitations amid rising diagnostic demand [42,43,44,45,46].

Beyond laboratory workflows, AI is increasingly applied across infectious disease management, supporting diagnostic decision-making, epidemiological analysis, and clinical risk stratification [47]. In addition, AI-driven approaches are increasingly applied in metagenomics, pathogen detection, and AMR surveillance, enabling integration of large-scale genomic and clinical datasets [48].

### 6.1. ML-Based AMR Phenotype Prediction from WGS Data

Early studies combining whole-genome sequencing with machine learning have established the foundation for genotype-based antimicrobial resistance prediction [49].

Supervised machine learning models trained on curated WGS datasets have demonstrated performance competitive with or superior to EUCAST/CLSI phenotypic breakpoints for genotype-to-phenotype AMR prediction in priority pathogens. A 2024 systematic review evaluating 26 studies integrating WGS and ML for AMR prediction in critical pathogens, encompassing 104,141 microbial samples, identified Random Forest (RF), XGBoost, and logistic regression as the highest-performing algorithms, with mean AUC values of 0.89, 0.87, and 0.87, respectively [40]. RF demonstrated superior performance with AUC values ranging from 0.66 to 0.97 across species–antibiotic combinations.

For Acinetobacter baumannii, one of the WHO critical priority pathogens, a 2024 study applying RF, SVM, and XGBoost with k-mer features from WGS data of 339 isolates predicted MICs for 13 antimicrobials, with category agreement exceeding 95.29% and essential agreement above 90.90%, demonstrating that ML-based AMR prediction can achieve interpretive agreement competitive with conventional AST [50]. For *Pseudomonas aeruginosa*, BioWeka and RF classifiers trained on WGS data achieved mean classification accuracy of 98% and 96%, respectively, across 12 antimicrobial classes [51].

Deep learning architectures have extended AMR prediction beyond resistance gene catalogs to capture complex genotype–phenotype relationships mediated by regulatory mutations, promoter polymorphisms, and epistatic interactions. A deep learning framework employing CNNs and denoising autoencoders for *M. tuberculosis* drug resistance prediction achieved a sensitivity of 0.90 and a specificity of 0.87 across first- and second-line antituberculars, and the model was made publicly accessible as TB-DROP [52]. The 2024 assessment of four state-of-the-art ML methods for AMR prediction across 78 species–antibiotic datasets highlighted that no single algorithm universally outperforms others, underscoring the need for pathogen- and antibiotic-specific model selection and validation [39].

Importantly, the performance of these models is inherently constrained by genotype–phenotype discordance, as discussed in Section 5, which may limit the accuracy of resistance prediction in complex or poorly characterized resistance mechanisms.

Despite these advances, significant challenges remain in translating AI-based AMR prediction into routine clinical practice, including data heterogeneity, model generalizability, and integration into diagnostic workflows [53]. Recent deep learning frameworks, including graph neural networks and transfer learning approaches, have further improved AMR prediction from genomic data [54,55]. AI-based approaches are also being explored for antimicrobial resistance management, supporting both prediction and optimization of therapeutic strategies [56].

### 6.2. AI-Assisted mNGS Interpretation

The clinical interpretation of mNGS data, distinguishing the infecting pathogen from colonizers, contaminants, or bystanders, remains a critical, unresolved challenge that AI approaches are beginning to address. Convolutional neural network and transformer-based models that integrate read counts, genome coverage breadth, phylogenetic signal, and patient clinical metadata have been developed to generate probabilistic pathogen likelihood scores that outperform abundance thresholding alone in discriminating clinically significant organisms from background [57]. The Karius Test, an FDA-authorized plasma mNGS platform, incorporates a proprietary AI interpretation module providing clinician-facing reports with pathogen-specific significance scoring, representing the most advanced commercially deployed AI-assisted mNGS diagnostic tool [58].

Importantly, AI models trained on single-center or geographically restricted datasets may exhibit significant performance degradation when applied to external populations with different pathogen epidemiology, microbiome composition, or specimen processing protocols—a generalizability challenge directly analogous to those documented in AI-based radiology and pathology applications. Multi-center AI model development and prospective external validation across diverse clinical settings are essential prerequisites for clinical translation [20].

### 6.3. Federated Learning and Data Governance

Federated learning—enabling ML model training across distributed healthcare datasets without centralized data sharing—offers a compelling solution to the data governance and patient privacy constraints that have historically limited multicenter AI development in clinical microbiology. Early implementations of federated AMR prediction models across multiple university hospitals have demonstrated model performance equivalent to centrally trained counterparts while maintaining full patient data sovereignty [59]. This approach is particularly relevant in the European regulatory context governed by the GDPR, where centralized aggregation of genomic data across institutions poses significant legal and ethical challenges.

While AI and machine learning approaches have demonstrated substantial potential in enhancing the interpretation of molecular diagnostic data, their clinical impact is ultimately realized through integration into real-world diagnostic workflows. In particular, translating AI-enabled analytical capabilities into decentralized and point-of-care (POC) settings represents a critical step toward clinically actionable implementation. The role of AI in supporting POC molecular diagnostics, including automated interpretation, quality control, and decision support integration, is further discussed in Section 7, where representative models and applications are summarized (Table 2).

## 7. Point-of-Care Molecular Diagnostics: From Laboratory to Bedside

The translation of molecular diagnostic capability to the point of care—the site of patient encounter without reliance on centralized laboratory infrastructure—is a strategic priority for infectious disease management, AMR stewardship, and pandemic preparedness [60]. The global POC infectious disease diagnostics market was estimated at USD 12–15 billion in 2024, with a compound annual growth rate of 8–9% projected through the late 2020s, driven by infectious disease assays and accelerated by innovations in molecular amplification, biosensors, microfluidics, and AI [60].

### 7.1. Commercially Available POC Molecular Platforms

The Cepheid GeneXpert system, the most extensively validated commercial POC molecular platform, encompasses cartridge-based assays for more than 20 bacterial and viral targets, including MRSA/SA, *C. difficile*, *N. gonorrhoeae/C. trachomatis*, *M. tuberculosis*/rifampin resistance, and *Streptococcus agalactiae*, validated across emergency departments, intensive care units, and resource-limited settings [61]. The study conducted by Emilio Bouza and colleagues demonstrates that integrating a rapid molecular diagnostic test such as GeneXpert MRSA/SA SSTI into a structured SSTI management program, combined with antimicrobial stewardship principles, significantly optimizes antimicrobial therapy. This approach enables the rapid identification of causative pathogens—particularly Staphylococcus aureus and MRSA—thereby facilitating early initiation of targeted treatment, reducing unnecessary use of broad-spectrum antibiotics, and ultimately helping to limit antimicrobial resistance. Furthermore, the implementation of such a “fast-track” program improves clinical workflow efficiency and may reduce hospital length of stay and overall healthcare costs [62]. The Abbott ID NOW platform employs isothermal amplification to deliver results in 5–13 min, while bioMérieux’s SpotFire MDx system is a next-generation POC platform that leverages novel approaches for broader pathogen target coverage [63].

The Frontiers 2026 synthesis of POC molecular technologies identified isothermal NAATs, rapid PCR, CRISPR-based detection, nanomaterial-enabled biosensors, and microfluidic platforms as the five principal technology classes advancing toward POC deployment, with all five achieving sub-30-min time to result in optimized formats [64]. Multiplex POC panels currently provide pathogen identification and resistance profiles in 45–90 min, enabling rapid, evidence-based therapy and minimizing unnecessary antimicrobial use—particularly valuable for MDR TB, drug-resistant *N. gonorrhoeae*, MRSA, and CRE [64].

### 7.2. Emerging POC Technologies: LAMP-on-Chip and CRISPR-POC

LAMP-based POC biosensors have seen substantial innovation in the 2020–2025 period. Colorimetric LAMP with pH-sensitive dyes enables visual interpretation of results without instrumentation, with LODs of 1–100 copies per reaction for bacterial targets [65]. Paper-based LAMP devices combining RNA extraction, isothermal amplification, and visual readout have achieved LODs of 10^3^ copies/mL with a time to result of 30 min [65]. Digital LAMP—partitioning reactions into thousands of microchambers for absolute quantification—extends LAMP’s analytical envelope toward the performance levels of ddPCR while preserving isothermal operation.

CRISPR-based POC diagnostics represent the technology with the strongest transformative potential for decentralized AMR diagnostics. CRISPR infrastructure investment requirements are estimated to be 90% lower than those for NGS, using lateral flow test strips or handheld devices [29]. CRISPR-Cas12a-integrated electrochemical biosensors (E-CRISPR) and isotropic electrophoresis technologies provide instrument-free POC solutions that do not require fluorescent labeling [32]. A portable Dragonfly LAMP-based platform demonstrated power-free nucleic acid extraction in under 5 min and colorimetric results in 30 min, with an LOD of 100 genome copies per reaction, and was validated across multiple pathogen targets in resource-limited settings [66].

### 7.3. WHO Target Product Profile Concordance and Implementation Gaps

The WHO Target Product Profile (TPP) for POC diagnostics in priority bacterial infections specifies minimum performance requirements of sensitivity ≥80%, specificity ≥95%, time to result <30 min, and cost per test <USD 5 for low- and middle-income country (LMIC) applicability [67,68]. Current commercial platforms meet sensitivity and specificity thresholds but substantially exceed cost thresholds, with most cartridge-based assays priced at USD 15–50 per test. Lyophilized LAMP reagents and CRISPR lateral flow assays are the most realistic near-term candidates for meeting WHO TPP cost requirements, with prototype costs of USD 2–5 per test in research-grade manufacturing contexts [29].

The implementation of POC molecular diagnostics in LMICs faces multidimensional barriers beyond reagent costs: cold-chain requirements for reagent stability, inconsistent electrical infrastructure, limited trained personnel, limited connectivity for result reporting, and the absence of post-market surveillance frameworks. Cloud-connected POC platforms with smartphone-based readout and AI-assisted interpretation are beginning to address connectivity and interpretive barriers, with digital health integration enabling near-real-time AMR surveillance linkage between clinical diagnostics and public health intelligence [64].

### 7.4. AI-Enabled POC Diagnostics

The integration of AI into POC molecular platforms, through edge-computing algorithms embedded in diagnostic devices and smartphone-connected cloud-based analytics, is enabling a new class of ‘smart’ POC diagnostics. These systems incorporate AI-driven capabilities, including automated result interpretation, signal classification, and real-time quality control, thereby reducing operator dependence and improving diagnostic reliability. In addition, AI integration facilitates connectivity with clinical and public health infrastructures, supporting real-time data transmission and antimicrobial resistance surveillance. The global POC diagnostics market, valued at approximately USD 53 billion in 2024, is expected to increasingly incorporate AI-enabled functionalities across molecular, biosensor, and microfluidic platforms [60]. Recent studies have demonstrated that deep learning-assisted predictive diagnostics, particularly those integrating time series AI architectures, can reduce assay interpretation time to 1–2 min in prototype infectious disease POC systems [69].

Taken together, these technological and analytical advances underscore the evolving landscape of point-of-care molecular diagnostics, where platform capabilities, implementation feasibility, and AI-enabled functionalities converge. A comparative overview of currently available and emerging POC molecular platforms, including their analytical performance and implementation characteristics, is presented in Table 3. The data summarized are derived from a narrative synthesis of the literature, integrating findings from primary studies, validation reports, and recent reviews. Given the variability in study design, specimen types, and deployment settings, reported values should be interpreted as representative ranges rather than directly comparable metrics.

## 8. Regulatory Frameworks and Health–Economic Considerations

The clinical implementation of molecular diagnostics is governed by jurisdiction-specific regulatory frameworks: FDA 510(k) or de novo authorization in the United States, CE-IVD marking under EU IVDR (Regulation 2017/746), and equivalent national frameworks in other jurisdictions. The EU IVDR—fully applicable from May 2022 with transition periods extending to 2027—has substantially raised the regulatory bar for in vitro diagnostics, requiring clinical evidence packages that many legacy molecular assays are only beginning to assemble [71].

Health–economic analyses of syndromic molecular panels for bloodstream infections have consistently demonstrated favorable cost effectiveness when structured antimicrobial stewardship programs are co-implemented. A cost effectiveness model of FilmArray BCID-guided stewardship in septic patients demonstrated an incremental cost effectiveness ratio of USD 4,200 per quality-adjusted life year gained versus conventional diagnostics plus standard stewardship—well within accepted willingness-to-pay thresholds [72]. For mNGS, a targeted deployment strategy—applied to patients with confirmed infection syndrome and culture-negative results after 48–72 h—achieves the highest cost–benefit ratio by concentrating diagnostic utility where incremental yield is greatest [73].

External quality assurance (EQA) participation through programs such as NEQAS Bacteriology, QCMD, and ECDC-coordinated AMR surveillance schemes is an essential component of infrastructure for maintaining molecular diagnostic quality standards across healthcare networks. Standardization of bioinformatic pipelines for WGS-based surveillance—actively promoted by ECDC through standardized core–genome MLST schemes and shared sequence repositories—remains a critical prerequisite for cross-border epidemiological comparisons and outbreak investigation [74].

## 9. Discussion

The rapid expansion of molecular diagnostic technologies has fundamentally reshaped the landscape of bacteriological diagnosis, enabling earlier pathogen detection, improved analytical sensitivity, and increasingly comprehensive AMR profiling. However, as highlighted throughout this review, no single platform achieves optimal performance across all clinically relevant dimensions, and important trade-offs persist among analytical speed, detection breadth, and interpretability.

Amplification-based methods, particularly qPCR and multiplex PCR panels, remain the most clinically mature technologies, offering rapid turnaround times and high sensitivity for targeted detection. Nevertheless, their inherent limitation in distinguishing viable from nonviable organisms introduces important challenges in clinical interpretation, especially in patients receiving antimicrobial therapy. This limitation underscores the need to contextualize molecular results within the broader clinical picture and, where appropriate, complement them with RNA-based or phenotypic approaches [16].

Sequencing-based platforms, including WGS and metagenomic NGS, provide unparalleled resolution for pathogen characterization and AMR profiling. Their integration into surveillance frameworks has enabled high-resolution tracking of pathogen evolution and transmission dynamics [20]. However, their clinical implementation remains constrained by cost, infrastructure requirements, and the complexity of data interpretation, particularly in distinguishing infection from colonization or contamination in metagenomic workflows [23].

Nanopore sequencing represents an important intermediate solution, combining rapid turnaround with genomic depth, and recent clinical studies have demonstrated its utility in real-time pathogen identification in bloodstream and central nervous system infections [24,25,26]. Despite these advances, variability in sequencing accuracy and the need for standardized analytical pipelines remain key barriers to routine clinical adoption.

CRISPR-based diagnostics have emerged as one of the most promising next-generation platforms, offering high sensitivity, single-nucleotide resolution, and compatibility with POC formats. However, as discussed, their translation into routine clinical practice remains limited by challenges related to assay standardization, scalability, and regulatory validation, highlighting the gap between analytical innovation and clinical implementation [32].

A central theme across all molecular approaches is the persistent gap between analytical detection and clinical interpretability. Genotype–phenotype discordance in AMR prediction remains a critical limitation, as the presence of resistance genes does not consistently translate into phenotypic resistance, and conversely, resistance may arise through mechanisms not captured by current molecular assays [18,38]. This limitation is particularly relevant for sequencing- and AI-based approaches, which rely on genomic data and may therefore inherit these discrepancies.

AI has emerged as a key enabler for addressing the complexity of molecular diagnostic data, particularly in interpreting WGS and mNGS outputs. AI-driven approaches have demonstrated substantial potential to improve diagnostic accuracy, automate data interpretation, and optimize laboratory workflows [42,43,44,45,46]. Moreover, the application of AI in AMR prediction, metagenomics, and clinical decision support reflects a broader shift toward data-driven infectious disease management [47,48]. However, important challenges remain, including model generalizability, data heterogeneity, and the need for robust external validation across diverse clinical settings [53].

The integration of molecular diagnostics into point-of-care settings represents a critical step toward reducing the diagnostic gap and enabling real-time clinical decision-making. While current POC platforms demonstrate promising performance, their implementation is influenced by cost, infrastructure, and regulatory constraints, particularly in low- and middle-income settings [67,68]. AI-enabled POC systems further extend this paradigm by enabling automated interpretation and connectivity with surveillance networks, although their real-world impact remains to be fully validated.

### 9.1. Limitations 

This review has several limitations. The analysis is based on a narrative synthesis of heterogeneous studies, which may limit direct comparability across platforms. In addition, the rapid pace of technological development means that recently introduced platforms, particularly in AI-assisted diagnostics and CRISPR-based detection, may already exceed the performance ranges summarized in this review.

Future research should focus on improving the standardization of analytical metrics, enhancing integration between molecular and phenotypic data, and validating emerging technologies in real-world clinical environments. Greater emphasis on clinical utility, rather than analytical performance alone, will be essential to ensure that advances in molecular diagnostics translate into improved patient outcomes and more effective antimicrobial stewardship.

### 9.2. Clinical Implications

From a clinical perspective, integrating molecular diagnostic technologies into routine practice has the potential to substantially reduce the diagnostic gap and enable earlier initiation of targeted antimicrobial therapy. Rapid amplification-based platforms and multiplex panels are particularly valuable in acute settings such as sepsis, where time to result directly influences patient outcomes. However, the limitations of molecular detection, including the inability to distinguish viable from nonviable organisms and genotype–phenotype discordance in AMR prediction, necessitate cautious interpretation and continued integration with phenotypic testing. The selection of diagnostic platforms should therefore be guided by clinical context, balancing speed, analytical depth, and interpretability to optimize patient management and antimicrobial stewardship.

### 9.3. Future Directions

Future developments in molecular bacteriological diagnostics are expected to focus on improving the integration of analytical performance with clinical interpretability. Advances in CRISPR-based platforms, AI-assisted diagnostics, and nanopore sequencing are likely to further enhance sensitivity, specificity, and real-time data analysis capabilities. In parallel, efforts to standardize performance metrics, expand resistance gene databases, and improve the validation of AI models across diverse clinical settings will be critical for clinical translation. The continued evolution of POC molecular systems, particularly in resource-limited environments, will depend on reducing costs, improving robustness, and ensuring interoperability with digital health and surveillance infrastructures.

## 10. Conclusions

Molecular diagnostic technologies have substantially advanced the detection and characterization of bacterial pathogens, offering rapid and sensitive alternatives to conventional culture-based methods. However, their clinical utility remains strongly dependent on diagnostic context, including pathogen type, specimen characteristics, and intended application.

While PCR-based approaches provide robust, rapid detection of well-characterized targets, sequencing-based methods enable comprehensive genomic insights, particularly for AMR profiling. Emerging technologies, including CRISPR-based diagnostics and AI-assisted analytical frameworks, further expand diagnostic capabilities, though their clinical integration remains under ongoing validation.

Importantly, molecular detection does not fully capture phenotypic expression, and limitations such as genotype–phenotype discordance and viability-related interpretability must be considered when translating analytical performance into clinical decision-making. Consequently, molecular diagnostics should be regarded as complementary to, rather than replacements for, phenotypic susceptibility testing in many clinical scenarios.

Overall, continued progress in molecular diagnostics will depend on improving integration with clinical workflows, ensuring robust validation across diverse settings, and aligning technological innovation with clinically meaningful outcomes.

## Figures and Tables

**Table 1:** Comparative analytical performance of major molecular diagnostic platforms for bacteriological diagnosis. Data synthesized from [8,9,10,11,12,13,14,15,16,17,18,19,20,21,22,23,24,25,26,27,28,29,30,31,32,33,34], representing aggregated performance metrics across multiple study periods and platform generations.

Platform	Sensitivity	Specificity	LOD	Time to Result	Multiplex Capacity	AMR Genotyping	POC Feasibility
qPCR/ RT-PCR	93–98%	97–99%	1–100 copies/reaction	1–3 h	Low (1–5 targets)	Targeted genes (mecA, vanA/B,blaKPC)	Moderate (GeneXpert)
Multiplex PCR Panel	90–97%	97–99%	10–100 copies/reaction	1–2 h	High (20–43 targets)	Panel-specific genes	Moderate (FilmArray)
Digital PCR (ddPCR)	98–99%	98–99%	0.1–1 copies/reaction	3–5 h	Low (1–3 targets)	High sensitivity variants	Low
LAMP	90–98%	97–99%	10–100 copies/reaction	15–60 min	Low (1–6 targets)	Selected resistance genes	High (colorimetric)
RPA	88–96%	95–99%	1–10 copies/reaction	10–30 min	Low	Selected targets	High
WGS (Illumina)	99%+	99%+	Not applicable (Requires prior culture)	24–72 h	Complete genome	Full resistome, MLST, plasmids	Very Low
mNGS	65–85% (clinical)	85–95%	Variable (depends on specimen type and sequencing depth)	12–48 h	Unlimited (hypothesis-free)	Full resistome (culture-free)	Very Low
Nanopore Sequencing	90–99% (targeted)	95–99%	~10 copies/reaction	1–6 h	High to complete	Full resistome, plasmid context	Low– Moderate
CRISPR (SHERLOCK/ DETECTR)	94–100%	98–100%	1–100 aM (analytical conditions)	30–60 min	Moderate (multiplex emerging)	SNV-level AMR variants	High (lateral flow)
CRISPR- Microfluidic	96–99%	99%+	1–10 copies/µL	30–45 min	High (8+ simultaneous)	AMR genes (emerging)	High (integrated chip)

LOD values are reported using different units (e.g., copies per reaction, copies per µL, or molar concentration) depending on the analytical methodology of each platform. Direct comparison across platforms should be interpreted with caution, as these values reflect different experimental conditions and input types (e.g., purified nucleic acid input versus clinical specimen matrices). Where applicable, values correspond to ideal analytical performance reported in the literature. LOD: limit of detection; AMR: antimicrobial resistance; MLST: multi-locus sequence typing; SNV: single-nucleotide variant; POC: point-of-care; aM: attomolar; rxn: reaction.

**Table 2:** Representative ML models for AMR prediction from WGS and phenotypic data. Data synthesized from [39,40,50,51,52,53,54,55,56].

Pathogen/Target	Algorithm	AUC/ Accuracy	AMR Classes Predicted	Dataset Size	Reference
A. baumannii (WGS)	RF + XGBoost + k-mer	CA >95.3%, EA >90.9%	13 antimicrobials	339 isolates	[50]
P. aeruginosa (WGS)	Random Forest/ BioWeka	98%/96% accuracy	12 classes	NR	[51]
Critical pathogens (WGS + AST)	RF, XGBoost, LR	AUC 0.89/0.87/0.87	Multiple	104,141 samples	[40]
M. tuberculosis (WGS + DL)	CNN + Denoising AE	Sens. 0.90, Spec. 0.87	First + second line	>38,000 genomes	[52]
AMR (phenotypic data)	XGBoost	AUC 0.96	Multiple organisms	917,049 isolates	[53,54,55,56]
E. coli/Salmonella WGS	Random Forest + k-mer	91–98% accuracy	Selected classes	Large-scale PATRIC	[39]

CA: category agreement; EA: essential agreement; AUC: area under the receiver operating characteristic curve; NR: not reported; DL: deep learning; AE: autoencoder.

**Table 3:** Comparative overview of POC molecular platforms for bacteriological diagnosis and AMR detection. Data synthesized from [32,60,61,62,63,64,65,66,69,70].

Platform	Technology	Time to Result	Sensitivity	Specificity	AMR Detection	LMIC Feasibility	Regulatory Status
GeneXpert (Cepheid)	Cartridge PCR	45–90 min	92–99%	97–99%	Yes (targeted genes)	Moderate	FDA/CE cleared
ID NOW (Abbott)	Isothermal (NEAR)	5–13 min	85–96%	98–100%	Limited	Moderate	FDA cleared
FilmArray (BioFire)	Multiplex PCR	45–90 min	93–97%	97–99%	Yes (panel-based)	Low (cost)	FDA/CE cleared
Colorimetric LAMP	Isothermal (LAMP)	15–45 min	90–98%	97–99%	Selected genes	High	CE (select)
CRISPR lateral flow	CRISPR-Cas12/13	30–60 min	94–100%	98–100%	SNV-level AMR	High (emerging)	CE pending
CRISPR-Microfluidic chip	CRISPR + microfluidics	30–45 min	96–99%	99%	AMR genes (emerging)	Moderate	CE validation
Nanopore POC (MinION)	Nanopore sequencing	30 min –6 h	90–99%	95–99%	Full resistome	Low	Research use
E-CRISPR biosensor	Electrochemical CRISPR	<45 min	95–99%	98–100%	AMR variants	High (emerging)	Pre-commercial

Data synthesized from multiple literature sources, representing aggregated performance and implementation characteristics across different study settings and platform generations. NEAR: nicking enzyme amplification reaction; LMIC: low- and middle-income country; SNV: single-nucleotide variant.

## Data Availability

No new data were created or analyzed in this study. Data sharing is not applicable to this article.
